# Seeds and the Art of Genome Maintenance

**DOI:** 10.3389/fpls.2019.00706

**Published:** 2019-05-31

**Authors:** Wanda M. Waterworth, Clifford M. Bray, Christopher E. West

**Affiliations:** ^1^University of Leeds, Leeds, United Kingdom; ^2^The University of Manchester, Manchester, United Kingdom

**Keywords:** DNA repair, seeds, germination, priming, aging

## Abstract

Successful germination represents a crucial developmental transition in the plant lifecycle and is important both for crop yields and plant survival in natural ecosystems. However, germination potential decreases during storage and seed longevity is a key determinant of crop production. Decline in germination vigor is initially manifest as an increasing delay to radicle emergence and the completion of germination and eventually culminating in loss of seed viability. The molecular mechanisms that determine seed germination vigor and viability remain obscure, although deterioration in seed quality is associated with the accumulation of damage to cellular structures and macromolecules including lipids, protein, and nucleic acids. In desiccation tolerant seeds, desiccation/rehydration cycles and prolonged periods in the dry quiescent state are associated with remarkable levels of stress to the embryo genome which can result in mutagenesis of the genetic material, inhibition of transcription and replication and delayed growth and development. An increasing number of studies are revealing DNA damage accumulated in the embryo genome, and the repair capacity of the seed to reverse this damage, as major factors that determine seed vigor and viability. Recent findings are now establishing important roles for the DNA damage response in regulating germination, imposing a delay to germination in aged seed to minimize the deleterious consequences of DNA damage accumulated in the dry quiescent state. Understanding the mechanistic basis of seed longevity will underpin the directed improvement of crop varieties and support preservation of plant genetic resources in seed banks.

## Background

Successful germination is a key developmental transition that is critical for plant propagation and is essential for both agriculture and the plant lifecycle. Modern farming requires high quality seed lots, with robust germination and seedling establishment that is tolerant of environmental stresses. In addition, programs for the *ex situ* conservation of plant genetic resources in seed banks are reliant on seeds and their properties, providing a lifeline to future generations. Both agriculture and plant conservation requires the maintenance of seed germination vigor and viability during storage. Recent work has shed light on the molecular aspects of seed longevity, revealing DNA repair mechanisms and the DNA damage response (DDR) as key factors which control germination and dictate the germination potential of a seed.

### Seed Germination

Seeds are propagules containing embryos in which growth is suspended. In this quiescent state, desiccation tolerant seeds, which represent the majority of plant species, exhibit a low moisture content (<15%) and repression of metabolic processes until rehydration occurs upon seed imbibition. Seeds that survive such low moisture contents are termed “orthodox” seeds, in contrast to those species incapable of withstanding such water loss which are termed “recalcitrant.” Orthodox seeds can remain viable in this dehydrated state for long periods of time, before being stimulated to germinate upon rehydration under favorable conditions for growth. Seeds exhibit considerable interspecific and intraspecific variation in longevity, and in many species can retain viability for decades. Remarkably, date palm seeds excavated from the archeological site of King Herod’s palace in Israel, were able to germinate after 2000 years (Sallon et al., 2008). Upon desiccation the cytoplasm transitions from a fluid to a glassy state which minimizes mobility of molecules and stabilizes cellular structures (Buitink and Leprince, 2008). The residual water in the desiccated seed is associated with biological molecules which provide resistance to freezing and formation of ice crystals. Seed germination is initiated by the imbibition of water by the seed and ends with the start of elongation of the embryonic axis and emergence of the radicle (Bewley and Black, 1994). Given an adequate supply of water, imbibition by the mature “dry” orthodox seed exhibits a triphasic pattern (Bewley, 1997). Phase I consists of water uptake that is largely a consequence of matric forces. In the mature seed, metabolism is reduced to very low levels, although all the components of a fully functional protein synthesizing system, including mRNA synthesized during the late stages of seed maturation are present in the quiescent embryo of a viable seed (Blowers et al., 1980). Within minutes of taking up water, imbibing seeds display rapid activation of respiratory and synthetic processes, *de novo* synthesis of protein and both ribosomal and messenger RNA along with mitochondrial ATP synthesis. Imbibition is followed by a lag phase (Phase II) in which water potential of the seed is in balance with its surroundings and there is no net water uptake. Phase III occurs as a consequence of radicle elongation and emergence that drives an increase in fresh weight. Both viable and non-viable seeds will exhibit phases I and II of water uptake but only viable seed are capable of entering phase III, which marks the completion of germination.

### The Importance of Seed Longevity

Seeds deteriorate with time and seed aging is exacerbated under suboptimal environmental and poor storage conditions such as high relative humidity and temperatures. In agriculture, high seed vigor, defined as rapid, uniform germination, and robust seedling establishment tolerant of adverse environmental conditions, is a major determinant of crop yields (Rajjou et al., 2012; Finch-Savage and Bassel, 2016). Low quality seed negatively impacts on final yield through reduced emergence, poor seedling establishment and reduced harvesting efficiency arising from non-uniformity of crop growth. Low vigor seeds germinate and establish poorly under stresses including low temperature, drought and anoxic waterlogged soils. Yield losses resulting from using low vigor seeds are further exacerbated as young seedlings are particularly vulnerable to environmental stresses such as drought, predation, pathogen attack, and weed competition (Finch-Savage and Bassel, 2016). The strong link between seed vigor and successful seedling establishment highlights the great potential for increasing crop yields through improved seed germination performance in the field (Powell and Matthews, 2012). Seed longevity is determined by the interplay of complex genetic and environmental factors (Clerkx et al., 2004; Joosen et al., 2012), and despite its economic, agronomic and ecological importance our current understanding of the molecular basis of seed longevity remains incomplete to date. Desiccation and rehydration cycles in combination with prolonged periods in a dry quiescent state are accompanied by reduced cellular maintenance activities and the progressive accumulation of damage to cellular ultrastructure and biological macromolecules including DNA, RNA, proteins and lipids (Powell and Matthews, 2012). Reactive oxygen species (ROS) produced during desiccation, storage and imbibition are an important causative factor of seed aging although significantly ROS also play critical roles as signaling factors that promote germination (Kranner et al., 2010). Consequently, desiccation tolerant seeds have evolved powerful protection and repair systems to minimize damage to cellular structures and biological molecules. Upon seed imbibition, cellular repair activities facilitate recovery from damage incurred during quiescence, and the speed and capacity for repair are closely linked to germination performance and the successful establishment of the young seedling (Powell and Matthews, 2012). The molecular factors which influence seed longevity have been recently reviewed (Sano et al., 2015). However, an expanding body of studies is defining the important link between repair mechanisms, germination and seed longevity, in particular the role of genome maintenance mechanisms, and will form the focus of this review.

### Factors Affecting Seed Vigor and Viability

The low metabolism of the quiescent embryo provides a barrier to repair activities, leading to the accumulation of macromolecular damage and seed aging. Suboptimal conditions during the late stages of seed development or during quiescence accelerate the deterioration of cellular components (Sattler et al., 2004). The increased requirement for repair leads to a delay to radicle emergence and reduced germination performance, ultimately resulting in failure to germinate and loss of seed viability. Seed aging not only delays radicle emergence but in many species leads to abnormal or weak seedlings (Powell and Matthews, 2012). Repair mechanisms reverse damage to cellular components, restoring cellular function prior to the initiation of growth post-germination. Genetic studies have identified the importance of pathways for cellular repair in maintaining the viability of the quiescent seed, as recently reviewed (Rajjou et al., 2012; Sano et al., 2015; Waterworth et al., 2015). The activity of these pathways influence seed longevity and there is evidence that plants are able to adapt to environmental changes to promote seed viability over a relatively short timescale (Mondoni et al., 2014).

### DNA Damage in Seeds

DNA, and the information it encodes, is irreplaceable if lost or degraded. DNA damage has immediate impacts on cellular function as DNA provides the template both for transcription and DNA replication. As meristems within the embryonic plant give rise to the mature plant, including the reproductive tissues, mutations incurred in seeds have the potential to be transmitted on to progeny (Ries et al., 2000). Accordingly, genome maintenance mechanisms in seeds are important not only for growth and development, but also in preserving the longer term stability of plant germplasm at the level of populations and species. Thus, DNA damage must be repaired early in imbibition prior to initiation of cell division, to maintain germination potential and minimize mutagenesis in subsequent seedling development (Waterworth et al., 2016). The requirement for extended repair of accumulated damage underlies the delay to germination characteristic of low vigor seed (Waterworth et al., 2010). In particular, seed aging is associated with progressive accumulation of DNA damage in the embryo, including increased levels of base loss, generating abasic sites, base modification, single strand DNA breaks (SSBs) and DNA double strand breaks (DSBs) (Cheah and Osborne, 1978; Dourado and Roberts, 1984a; Córdoba-Cañero et al., 2014). For example, naturally aged rye seeds accumulated DNA breaks as seeds deteriorated, leading to prolonged DNA repair synthesis prior to the onset of DNA replication in aged seed and germination coincident with delayed radicle emergence (Cheah and Osborne, 1978; Elder et al., 1987). The lowered moisture content of the desiccated orthodox seed reduces the rate of genome damage but in the absence of repair, lesions accumulate over time (Walters et al., 2006). Desiccated maize seed incurred 6-fold less base loss after dry storage at 20°C for 2 years than DNA in aqueous solution. Apurinic (abasic) sites were detected at a frequency of 3.8 × 10^-5^ per nucleotide in the quiescent embryo, and levels further increased 4-fold upon imbibition (Dandoy et al., 1987). DSBs are a particularly cytotoxic form of DNA damage. Cytological studies demonstrated extensive chromosome fragmentation and rearrangements upon seed aging and that even high vigor seeds display a background level of DSBs (Dourado and Roberts, 1984a). An early study published by Navashin in 1933 reported that the incidence of chromosome abnormalities in a seed lot stored for a number of years “*…strikingly resembled one obtained from soaked seed which had been treated by X-rays*” (Navashin, 1933), with extensive chromosomal defects in the majority of cells. The aberrant mitotic figures represent mis-joined chromosomes resulting from extensive induction of DNA double strand breaks in the desiccated quiescent seed (Waterworth et al., 2016). Desiccation as a strategy to survive extreme environments is termed anhydrobiosis, and is found in a broad range of organisms including bacteria, tardigrades, fungi, algae and mosses (Franca et al., 2007). Desiccation tolerance requires protection adaptions, for example the production of late embryogenesis abundant (LEA) proteins which were initially identified in plants but found in diverse phyla. However, while protective mechanisms enable organisms to withstand the physical effects of dehydration and rehydration alone, they are insufficient or unable to counter the accumulation of DNA damage during quiescence. Several organisms adapted for anhydrobiosis have evolved powerful DNA repair mechanisms to reverse genome damage incurred during quiescence. Examples include tardigrades and the desert dwelling bacterium *Deinococcus radiodurans*, organisms that exhibit extreme resistance to high energy irradiation (X-rays and gamma rays) due to their enhanced DNA repair capacity (Zahradka et al., 2006; Gladyshev and Meselson, 2008).

### Genome Maintenance Mechanisms

**Figure 1:**
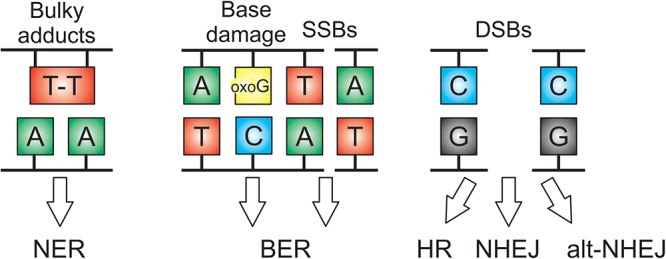
DNA damage lesions and their DNA repair pathways in seeds. Nucleotide excision repair (NER) repairs damage on a single strand of the duplex, with specificity for bulky adducts and forms of damage that block RNA polymerase. Base excision repair (BER) removes damaged bases and repairs single strand breaks (SSBs). DNA double strand breaks (DSBs) are repaired by homologous recombination (HR), non-homologous end joining (NHEJ), or alternative NHEJ. Oxo G is 8-oxoguanine.

The combination of endogenous factors and environmental stresses, including UV, background irradiation, soil and air pollutants, result in a wide spectrum of DNA damage. Furthermore, DNA modification by metabolic by-products (in particular ROS) and errors during DNA replication and transcription represent major sources of genome damage. Eukaryotes have evolved powerful DNA repair pathways specific for particular types of lesion (Figure 1) and sensitive DNA damage sensing mechanisms coupled to checkpoints that delay cell cycle progression in the presence of DNA damage (Sancar et al., 2004). Cellular survival depends on the concerted action of powerful repair pathways for base damage and single strand breaks (base excision repair or BER), broad specificity repair of damage to one strand of the duplex (nucleotide excision repair or NER) and repair of DNA double strand breaks (non-homologous end joining or NHEJ, homologous repair or HR, alternative NEJ pathways or alt-NHEJ). These pathways are highly conserved across eukaryotes, and well-characterized in plants, in particular in *Arabidopsis* and rice (Britt, 1999; Bray and West, 2005).

#### Excision Repair Pathways

Excision repair operates on one of the two strands of the DNA duplex by excising the damaged region followed by repair synthesis using the intact template strand as a guide. Damaged bases are typically removed by the Base Excision Repair pathway, initiated by DNA glycosylase enzymes that are specific to particular damage products, generating an abasic site which is followed by removal of the abasic site and DNA synthesis to fill the resulting gap. The most prevalent form of base damage is the oxidation product 8-oxoguanine (8-oxoG) and levels increase in seed subject to accelerated aging (Chen et al., 2012). Removal of 8-oxoG is mediated by either the 8-oxoguanine DNA glycosylase/lyase (OGG1) or formamidopyrimidine-DNA glycosylase (FPG) (Córdoba-Cañero et al., 2014). Both *OGG1* and *FPG* display increased expression during *Medicago truncatula* seed imbibition (Macovei et al., 2011) and levels of 8-oxoG base damage were significantly reduced in *Arabidopsis* seeds overexpressing *OGG1* (Chen et al., 2012). These lines also displayed enhanced resilience to seed aging under abiotic stress conditions, with improved seed viability when germinated at elevated temperatures or in the presence of salt stress (NaCl), relative to wild type (Chen et al., 2012). More bulky forms of DNA damage, representing steric changes in DNA duplex structure including base dimers, are repaired by nucleotide excision repair (NER), in which an oligonucleotide of ∼30 bases is excised and DNA polymerase fills in the single stranded region. This pathway can also use stalled RNA polymerase to identify polymerase blocking lesions which are then fed into the NER pathway. Mutation in xeroderma pigmentosum group B protein (XPB1), which mediates DNA helicase activity in NER, resulted in reduced germination relative to WT seeds after treatment with hypochlorite, which induces oxidative DNA damage. This suggests that NER is active in imbibing seeds and is required for maintenance of seed viability (Costa et al., 2001). NER gene expression increased toward the end of *Phaseolus vulgaris* L seed development, consistent with NER activity in imbibing seeds (Parreira et al., 2018). To-date there are no reports that core NER components are required to repair aging-induced genome damage, although recently co-expression network analysis in *Medicago* and *Arabidopsis* identified DNA repair factors such as *DNA LIGASE I* (*LIG1*) as genes associated with seed longevity (Righetti et al., 2015). Genes in this cluster were also expressed in response to pathogens, light and auxin, raising the possibility that seed longevity may have evolved through co-opting pathways which control defense against pathogens (Righetti et al., 2015). Interestingly the DDR signaling network is common to a broader range of stresses and has been implicated in the response to pathogen attack (Ogita et al., 2018).

#### Repair of DNA Double Strand Breaks

Double-strand breaks are highly cytotoxic DNA damage products which occur spontaneously in the cell, especially during DNA replication and under oxidative stress. DSBs are repaired by non-homologous end joining (NHEJ) or homologous recombination (HR), characterized by random-end-joining or homology mediated repair of broken chromosomes, respectively. NHEJ is the predominant mechanism in vegetative tissues of vascular plants, as indicated by the extreme hypersensitivity of NHEJ mutants to X-rays and radiomimetics (West et al., 2002; Friesner and Britt, 2003). Recombination-mediated repair of DSBs is essential for cell viability and maintenance of genomic integrity in response to genotoxic stresses (Charbonnel et al., 2011). The elevated frequencies of chromosomal abnormalities in aged seeds (Abdalla and Roberts, 1968) arise from chromosomal fusions formed through errors in re-joining of DNA DSBs by the cell’s recombination pathways. Even high vigor seeds display a background level of chromosomal aberrations, indicative of higher levels of genome stress in germination relative to other stages of plant development (Waterworth et al., 2016). In *Arabidopsis* seeds, the presence of chromosomal breaks is sufficient to slow or block germination and failure to repair this damage prior to germination results in genome instability and low vigor seedlings (Waterworth et al., 2010). Analysis of DNA ligase mutants, deficient in NHEJ repair of DSBs, established the genetic link between DNA repair and seed longevity. DNA LIGASE 4 (LIG4) and DNA LIGASE 6 (LIG6), respectively, function in the canonical and back-up (alt-NHEJ) pathways, and mutant seed are hypersensitive to accelerated aging (Charbonnel et al., 2011). The additive phenotype of the *lig4 lig6* mutant indicates distinct roles for each pathway in maintenance of germination potential. Interestingly, a genome wide analysis of genetic determinants of seed longevity identified a QTL in *Arabidopsis* that coincided with the chromosomal location of *LIG4* (Nguyen et al., 2012). HR-mediated repair of DSBs is also important in seeds, identified by analysis of gamma irradiated maize *rad51* mutants which displayed delayed germination and high seedling mortality relative to wild type lines (Li et al., 2007). The hypersensitivity to aging of seeds deficient in DSB repair implicates the importance of chromosome break repair in maintaining high seed vigor (Waterworth et al., 2010). Conversely, increased DNA repair capacity results in enhanced seed longevity and resistance to aging (Chen et al., 2012) and seeds that are maintained in a hydrated state and which have not undergone maturation drying do not display such levels of genome stress, with reduced chromosomal abnormalities (Villiers, 1974). During the later stages of seed development in *Phaseolus vulgaris*, in which maturation drying reduces seed water content, seeds display upregulation of DSB repair associated genes, which may reflect the stress induced during the drying phase and may prime seeds with repair factors required in early imbibition (Parreira et al., 2018). These results establish a strong link between DNA damage incurred during seed aging with decreased seed quality and weak seedlings that establish poorly on soil.

### DNA Damage Signaling

**Figure 2:**
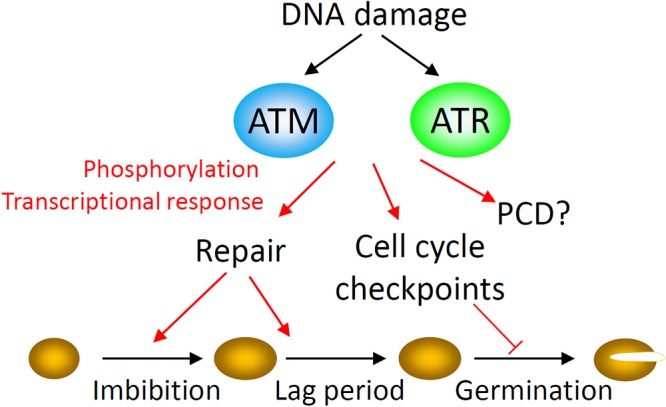
The DNA damage response (DDR) in seeds. The DNA damage response executes a coordinated network of responses in order to minimize the consequences of genome damage to the cell, including activation of cell cycle checkpoints, DNA repair factors, and programmed cell death (PCD). The master kinases ATAXIA TELANGIECTASIA MUTATED (ATM) and ATM AND RAD3-RELATED (ATR) control the cellular response to DNA damage in eukaryotes through activation of downstream responses at the transcriptional and post-transcriptional levels. ATM controls advancement of germination in aged seeds, in part through transcriptional control of the cell cycle inhibitor *SIAMESE RELATED 5* (*SMR5*). Both ATM and ATR influence seed viability but the molecular mechanism is unknown. In plants the transcriptional DDR encompasses hundreds of genes encoding proteins involved in DNA repair, chromatin remodeling and DNA metabolism. In the early stages of imbibition, seeds exhibit a large and rapid ATM-dependent transcriptional DNA damage response early in imbibition. DNA repair synthesis is detectable from the earliest stages of imbibition. As seed aging progresses and radicle emergence is delayed, this lag phase to germination is accompanied by an ATM-mediated delay of cell cycle activation in the root apical meristem (RAM) and extension of DNA repair activities.

DNA damage sensing mechanisms are coupled to control of cell cycle progression to limit the potentially highly mutagenic effects of DNA replication or chromatid segregation in the presence of DNA damage (Sancar et al., 2004). In order to minimize the cellular consequences of genotoxic stresses, the DDR orchestrates a coordinated network of responses including activation of cell cycle checkpoints, DNA repair factors, programmed cell death (PCD) and endoreduplication (Fulcher and Sablowski, 2009; Adachi et al., 2011). Plant growth and development requires cellular responses to genotoxic stress, which are type-specific and dependent on damage levels (Fulcher and Sablowski, 2009). The protein kinases ATAXIA TELANGIECTASIA MUTATED (ATM) and ATM AND RAD3-RELATED (ATR) function as master controllers of the cellular response to DNA damage in eukaryotes and cell cycle arrest is activated, in part, by the transcriptional upregulation of CYCLIN DEPENDENT KINASE (CDK) inhibitors (Figure 2) (Yi et al., 2014; Hu et al., 2016). In plants the transcriptional DDR encompasses hundreds of genes encoding proteins involved in DNA repair, chromatin remodeling and DNA metabolism (Culligan et al., 2006). In the early stages of imbibition, seeds exhibit a large and rapid ATM-dependent transcriptional DDR, indicative of high levels of genotoxic stress (Waterworth et al., 2010). However, the DDR is negligible in mature barley seeds which have not undergone desiccation, storage and rehydration, indicating that ATM activation during imbibition of the desiccated seed is a direct response to high levels of DNA damage incurred during or after maturation drying (Waterworth et al., 2016). Recent studies identified that aged mutant *atr* and *atm* seeds display higher germination rates than wild type control seed, indicating deficiencies in the regulation of germination in response to damage in these lines (Waterworth et al., 2016). In ATM-deficient seeds, germination of aged seeds coincides with extensive chromosomal abnormalities and the resulting seedlings establish poorly on soil (Waterworth et al., 2016). Similarly, natural loss of seed vigor is associated with increased frequencies of non-viable seedlings carrying cytogenetic defects and leads to reduced crop yields in agricultural species (Dourado and Roberts, 1984b; Finch-Savage and Bassel, 2016). These recent findings collectively implicate DNA damage checkpoints as important determinants of vigor and viability of both seeds and seedlings, highlighting the importance of DNA damage signaling in germination to promote robust seedling growth.

### Cell Cycle Activity in Germination

An increasing body of studies is linking control of cell cycle in germination with seed vigor. Cell cycle progression is linked to genome integrity through the activity of cell cycle checkpoints which control cell cycle advancement. Checkpoints are activated at critical phases of the cell cycle including DNA replication (the G1/S transition and intra-S phase), and before partitioning of sister chromatids into daughter cells during mitosis (the G2/M checkpoint) (Hu et al., 2016). Advancement through the plant cell cycle is driven by CDKs (cyclin dependent kinases) and their regulatory cyclin partners and is stimulated by CDK activating kinases (CAKs). Negative regulators integrate environmental and developmental signaling to control cell cycle activity. These include the WEE1 kinase, involved in the S-phase checkpoint, and two families of small inhibitory proteins: cyclin-dependent kinase inhibitors (CKIs, also known as kip-related proteins (KRPs) and the SIAMESE/SIAMESE RELATED (SIM/SMR) family proteins (Hu et al., 2016). In most mature desiccated seeds the majority of cells are in the resting or G1 stage of the plant cell cycle (Velappan et al., 2017). Cell expansion drives embryo growth in *Arabidopsis* and the cell cycle is initiated in the cells of the root apical meristem (RAM) around the time of radicle emergence from the seed coat (Vázquez-Ramos and de la Paz Sánchez, 2007). Recent studies showed that phytohormones including gibberellin and auxin promote cell cycle activation prior to germination (Lara-Núñez et al., 2008; Resentini et al., 2015; Godínez-Palma et al., 2017), whereas activation of cell division in the cotyledons and shoot apical meristem (SAM) occurs largely post-germination, several hours later than the root meristem cells (Barroco et al., 2005; Masubelele et al., 2005). Cell cycle activity in the RAM is required for high vigor and there is evidence for regulatory roles of cyclins, KRPs, and SMR proteins in seed germination. Mutants lacking the D-type cyclins, CYCD1:1 and CYCD1:4 exhibited delayed radicle emergence (Barroco et al., 2005; Masubelele et al., 2005), while CYCD-CDK kinase activity in imbibing maize seeds is stimulated by auxin (Lara-Núñez et al., 2008).

Endocycles, whereby cells replicate DNA and increase ploidy without mitotic division, is associated with cell expansion and in seeds is implicated in stimulating germination (Finch-Savage and Bassel, 2016). For example, the *Arabidopsis* CDK inhibitor ICK3/KRP5 is expressed in the transition zone between the root and the hypocotyl. Mutants display delayed germination, consistent with a role for KRP5 in the induction of endocycles promoting radicle emergence (Wen et al., 2013). KRP6 is also suggested to promote germination through endocycles. However, KRP6 has additional inhibitory roles, counteracting the gibberellin-mediated activation of mitotic cell cycle activity, leading *krp6* mutants to germinate faster than wild type lines (Resentini et al., 2015). Recently, the second family of small inhibitory proteins (SIM/SMR) was also shown to have roles in seeds. In response to DNA damage, *Arabidopsis* ATM induces expression of *SMR5* and *SMR7* which results in cell cycle arrest (Yi et al., 2014) and *SMR5* and *SMR7* induction is also observed during imbibition (Waterworth et al., 2016). This is indicative of a mechanism whereby DNA damage slows germination through the ATM-dependent cell cycle regulation mediated by SMR factors. Recent studies identified that in aged seeds of *atm* mutant lines, S-phase is advanced relative to wild type seed, consistent with ATM-mediated control of a G1/S checkpoint and extending the lag period to completion of germination. This reveals DNA damage signaling as a major factor which controls germination in aged seed, integrating germination progression with surveillance of genome integrity and imposing the lag period to germination as vigor declines in response to aging-related DNA damage.

### Cell Death in Aged Seeds

Rapid and sensitive responses to genotoxic stresses are crucial to safeguard the fidelity of genetic information, in particular in meristematic tissues of plant embryos where actively dividing cell populations are the progenitors of all cells in the future plant. The genome integrity of the meristem cells, and especially the stem cell initials and the quiescent centre (QC), is therefore crucially important. The slow division rate of QC cells allows greater time for repair of genome damage and may underlie the greater tolerance of these cells to DNA damage (Heyman et al., 2014). This contrasts with the rapidly dividing stem cell initials which display hypersensitivity to genotoxins, leading to high levels of PCD in these tissues, in a pathway dependent on DNA damage signaling by the ATM and ATR kinases (Fulcher and Sablowski, 2009; Furukawa et al., 2010). Both kinases act through a transcription factor SOG1 which is proposed to have key roles in the resumption of embryo growth in germination subsequent to genome damage (Johnson et al., 2017). In seeds, cells remain in G1 prior to germination and the role of PCD and its contribution to seed vigor and subsequent seedling growth is unclear. However, hallmarks of cell death are observed as seed deterioration progresses and damage to cellular components exceeds repair capacity (Kranner et al., 2010). The appearance of DNA laddering, a characteristic hallmark of programmed nuclease activity in PCD, was detectable in both sunflower and pea seeds after aging, increasing in incidence as seed deterioration progressed (El-Maarouf-Bouteau et al., 2011; Chen et al., 2013). Transcriptomic analyses of aging pea seeds were consistent with a switch from PCD to senescence associated gene expression as seed viability is lost in pea. PCD in aged seeds may contribute to loss of viability, in addition to cell death arising from senescence of cells suffering irreversible damage, leading to “exhaustion,” which is likely to underlie the loss of germination potential in aged seeds (Kranner et al., 2010).

### Chromatin Remodeling

DNA repair, DNA replication and transcription all take place in the context of chromatin in which DNA is packaged with histone proteins into nucleoprotein complexes. Accessibility of proteins, including the transcription and repair machinery, is achieved through chromatin remodeling enzymes and post-translational modification of histones (Donà and Mittelsten Scheid, 2015). This provides a powerful mechanism for transcriptional control during development and in response to the environment, in addition to protecting DNA from cellular factors. The mechanisms which function to stabilize and protect the genome in the dry quiescent state, and in transitions in nuclear architecture between the hydrated and desiccated state, are unclear (Neumann et al., 2009). In *Arabidopsis* seeds, a programmed decrease in nuclear size and chromatin compaction are associated with the dormant and the desiccated state and persist until germination is completed (van Zanten et al., 2011). Chromatin remodeling plays important roles in the modulation of dormancy, which represents a block to germination even under favorable conditions, and recent studies are also revealing key roles in germinating seeds. Treatment of *Arabidopsis* seeds with histone deacetylase inhibitors stimulates germination (Wang et al., 2016), while *Arabidopsis* mutants in the histone deacetylases HDA6, HDA9 and HDA19 displayed reduced dormancy (Zanten et al., 2014; Nelson et al., 2017). HDA19 functions in a complex with SWI-INDEPENDENT3 (SIN3)-LIKE1 (SNL1) and SNL2 during seed maturation, and establishes seed dormancy through reducing expression of genes involved in ABA turnover, thereby promoting ABA accumulation (Wang et al., 2013). Upon imbibition, SNL1 and SNL2 expression is reduced, which results in increased histone acetylation in target genes and leads to auxin signaling, increased expression of CYCD1;1 and CYCD4;1 and promotion of germination (Wang et al., 2016). Deacetylation inhibitors, used at higher concentrations than those that increased *Arabidopsis* seed vigor, inhibited *Medicago* seed germination and resulted in increased DNA strand breaks around the time of radicle protrusion (Pagano et al., 2018). This DNA damage was coincident with upregulation of transcripts encoding antioxidant genes and the DNA repair factors OGG1 (BER) and LIG4 (NHEJ) (Pagano et al., 2018). Understanding how nuclear compaction is mediated with local changes in chromatin structure, and the impact of these modifications on germination and maintenance of genome integrity in the desiccated state, will provide important new insight into the mechanisms underlying seed longevity.

### Germination Enhancement Treatments: Seed Osmopriming

Deterioration in seed vigor is manifest as decreasing rapidity and synchronicity of germination and this increased delay to radicle emergence is accompanied by an extended period of genome repair. Several crop species, including high value vegetable seeds and sugar beet are routinely improved by priming, a pre-germinative seed treatment in which controlled hydration increases the speed of germination and enhances field emergence (Heydecker et al., 1973). Controlled hydration is thought to allow cellular repair processes to proceed without completion of germination (Heydecker et al., 1973; McDonald, 1999). Priming evidently reverses the lag period to germination exhibited as seed lose vigor and promotes uniformity and stress tolerance in emerging seedlings. Seedling field emergence for many commercial species, typically >70% in the case of sugar beet, can be increased 5–10% by priming. The advantages of priming treatments are reductions in both the spread of germination and mean time to germination in low vigor seed lots. However, priming can result in a significant reduction in seed longevity (accelerated loss of viability over time) resulting in substantial economic losses in crop species (Tarquis and Bradford, 1992; Dekkers et al., 2015). The molecular basis for this loss of storability remains unknown, although over-priming, where germination is allow to progress to the initiation of DNA replication, was associated with reduced viability in tomato (van Pijlen et al., 1996).

### Biochemistry of Osmopriming

Our understanding of the molecular basis of priming remains limited, although storage protein mobilization, endosperm weakening and DNA repair synthesis have been identified in a number of priming studies (Capron et al., 2007; Waterworth et al., 2015). Restoration of genome integrity by repair processes is common to priming in a range of species, including a correlation of DNA repair synthesis with improved germination after priming leek (*Allium porrum* L.) seeds (Ashraf and Bray, 1993; van Pijlen et al., 1996). Both repair of nuclear DNA and replication of mitochondrial DNA were observed during the priming period in leek embryos, whereas nuclear replicative DNA synthesis and cell cycle progression occurred post priming. Repair of mitochondria is likely to be of critical importance, as ATP is virtually absent in the quiescent embryo and mitochondrial oxidative phosphorylation is a major source of ATP from the start of imbibition. Loss of vigor has been shown to be reflected in reduced levels of nucleoside triphosphates and nucleotide sugars needed for nucleic acid synthesis and repair along with cell wall synthesis during cell expansion and division in the embryo of the germinating seed (Standard et al., 1983). Nuclear DNA replication is not observed during priming of leek seeds (Gray et al., 1990b). However, cell cycle progression during osmopriming treatments is species dependent and seeds of some species contain immature embryos which need to increase appreciably in size before the radicle tip emerges through the seed coat at germination. Such immature embryos of both carrot and celery seeds show a 3–4 fold increase in cell number and cell volume before they are able to germinate (Gray et al., 1990a; Karssen et al., 1990). During priming cells of the root tip of tomato embryos progress from the G1 phase of the cell cycle into G2 via a round of replicative DNA synthesis but do not undertake cell division (Bino et al., 1992; Dawidowicz Grzegorzewska, 1997; de Castro et al., 2000), consistent with cell cycle activity contributing to the advancement of germination conferred by seed priming.

### Genome Maintenance in the Hydrated Seed

In the natural environment, seeds can persist in the soil seedbank undergoing dormancy cycling for many years, experiencing transitions between wet-dry states dependent on soil hydration levels (Footitt et al., 2011). Seed-bearing plants are thought to have evolved dormancy and desiccation tolerance as distinct adaptive strategies which facilitate survival and propagation in varying environments, with many species exhibiting interspecific in addition to intraspecific adaptation to different climatic conditions (Nguyen et al., 2012; He et al., 2014). In the dormant hydrated state, genome maintenance activities reverse cellular damage accumulated in the desiccated state (Elder and Osborne, 1993), potentially reducing the acute requirement for DNA repair during germination observed in imbibing seeds. The negative correlation between seed dormancy and longevity indicated that repair capacity may be linked to the ecological niche that a species is adapted to Nguyen et al. (2012). Thus, seeds from dry environments may have lower dormancy but a greater requirement for cellular repair, resulting in enhanced longevity, whilst wetter environments support continuous background levels of cellular repair, but require greater control in the timing of germination. DNA repair synthesis is observed in hydrated, dormant wild oat seeds (*Avena fatua*) which initiate DNA replication only after transfer of seeds to temperatures permissive of germination (Elder and Osborne, 1993). Recent studies identified that *Arabidopsis* seeds display significant upregulation of mRNA transcripts of genome maintenance factors, including *LIG6*, *SMR5* and *ATM*, during prolonged hydration in the dormant state, consistent with repair activity in the soil seed bank (Waterworth et al., 2016). Notably, dormant, hydrated lettuce seed sustained less chromosomal damage and retained germination vigor for extended time periods in comparison to their dry stored counterparts (Villiers, 1974). DNA repair activities in desiccation-rehydration cycles has also been identified which functions to help maintain Artemesia seed viability in harsh desert conditions. These seeds contain a water-absorbing proteinaceous surface pellicle and the partial hydration of this pellicle by night-time desert dews was correlated with significant DNA repair activity serving to maintain the integrity of the embryo genome (Yang et al., 2011). Genome maintenance is required to minimize the mutational load as seeds deteriorate and germination vigor is lost (Waterworth et al., 2015). The spectrum of mutations incurred upon seed aging can be transmitted to future generations, with the potential to influence plant genome stability at the population level (Ries et al., 2000; Jiang et al., 2014). Seeds of wild populations are particularly sensitive to environmental perturbation (Cochrane et al., 2011) and stresses experienced at this stage of the plant life cycle may have significant impact on genome stability.

### Homeostasis of Reactive Oxygen Species in Seeds

Oxidative stress is a major cause of DNA damage, although oxidation of macromolecules is associated with both promotion of germination through ROS-mediated signaling in addition to the accumulation of oxidative damage as seeds deteriorate (Kranner et al., 2010). Oxidative stress activates components of the plant DDRs through ATM kinase signaling (Yi et al., 2014). In other eukaryotes ATM acts as a direct sensor of oxidative stress, although the mechanism of ATM activation is not reported in plants. Levels of ROS in seeds are controlled by non-enzymatic ROS scavenging systems and antioxidant enzymes such as peroxidases (catalase, peroxiredoxins), superoxide dismutase, and enzymes of the glutathione and ascorbate cycles (Bailly, 2004; Kranner et al., 2010; Sano et al., 2015). In wheat seeds, the peroxidase 1-cys peroxiredoxin (PER1) forms part of a nuclear-localized redox system (Pulido et al., 2009). Recently, ectopic expression of PER1 from sacred lotus, a species with extreme seed longevity, was shown to confer resistance to *Arabidopsis* seed aging, accompanied by reduced levels of ROS and lower lipid peroxidation (Chen et al., 2016). Lotus PER1 reduces Fe^3+^ mediated cleavage of plasmid DNA *in vitro*, and this activity together with nuclear localisation of this redox factor, provides a potential mechanisms for the protection of the seed genome.

### Combinatorial Consequences of Seed Deterioration

All the components of a fully functional protein synthesizing system, including messenger RNA, are present in the dry embryo of seeds. Viable embryos require only the imbibition of water for activation of metabolism and *de novo* protein synthesis is detectable within minutes of imbibing water in viable embryo (Bewley and Black, 1994). Germination is associated with massive transcriptional reprogramming as stored transcripts associated with seed maturation and quiescence are degraded in early imbibition and replaced by *de novo* synthesis of mRNA species required for seedling growth (Rajjou et al., 2004). DNA repair synthesis is initiated very early upon seed imbibition with the first burst of metabolic activity (Elder and Osborne, 1993). This is suggestive that at least some DNA repair factors may be either stored in the quiescent seed and become activated upon imbibition or produced by *de novo* synthesis upon resumption of transcription/translation (Elder and Osborne, 1993). During seed deterioration, damage to DNA, RNA, and protein progressively accumulates, increasingly impacting on the efficiency of transcription and translation processes in germination and early seedling growth. An important consequence of the requirement for *de novo* protein synthesis in germination is that seeds must preserve the translation machinery, as if it inactivated the capacity for production of replacement proteins becomes limiting (Rajjou et al., 2008; Dirk and Downie, 2018). Protein oxidation and mis-folding will also impact on efficiency of enzyme activities, including those of DNA repair factors such as DNA ligase and DNA polymerase, which decline in activity as seeds near the viability threshold (Elder et al., 1987; Gutiérrez et al., 1993; Coello and Vázquez-Ramos, 1996). However, the temporal progression of DNA damage signaling and repair processes in germination and how these are affected during seed aging largely remains to be determined.

### Future Questions

Recent studies have implicated important roles for DNA damage signaling in control of germination in the aging seed. However, how DNA repair processes and the DNA damage signaling networks are integrated with other key regulatory factors which control germination, dormancy and seed longevity remains to be established. Additionally, the genome maintenance mechanisms operative in dormancy and priming remain to be defined at the molecular level. The plant DDR is a complex signaling network with hundreds of downstream targets which orchestrates the cellular response to DNA damage (Culligan et al., 2006). Although ATM controls progression of germination in part through control of cell cycle activation in the RAM (Waterworth et al., 2016), further targets of DNA damage signaling and their functions remain to be determined. Furthermore, the contribution of DNA damage and roles of the DDR in loss of seed viability remains unknown. Future work will uncover these signaling pathways and provide an understanding of how germination is linked to genome integrity, with the identification of specific regulatory mechanisms and the cells and tissues of the plant embryo in which they operate. This will include analysis of agronomically important species, enabling the prediction and improvement of germination under stress conditions, through marker assisted breeding and utilization of intraspecific variation. Germination potential is also important to natural ecosystems, and defining the repair activities in seeds undergoing wet- dry cycling cycles in the soil seed bank will provide new insight into how genome integrity is preserved during environmental stresses. While repair factors are important, understanding the roles of chromatin remodeling, antioxidant systems and cellular protective factors in maintenance of germination potential will also help both understand and improve seed longevity.

## Conclusion and Outlook

The use of seeds for crop production was central to the development of human civilisation, underpinning agriculture and food production from Neolithic times until the present day. The increased demand for food with growth of the global population is leading to escalation in the value of the commercial seed market, projected to reach $92 billion by 2025 (Anon, 2019). Additional pressures on global agriculture result from the reduction in arable land, changing climate and the rising demand for biofuels. These factors necessitate the development of improved crop varieties that are tolerant of suboptimal environmental conditions and reduced losses arising from poor germination and field establishment. The escalating global population places enormous pressure on the environment, threatening many species with extinction. This has led to programs for plant germplasm conservation in seed banks, reliant on the storage properties of seeds. Both agriculture and plant conservation requires the maintenance of seed viability during storage, and recent work is shedding light on the molecular aspects of seed longevity, including key factors that dictate the germination potential of a seed. The seed stage of the plant lifecycle is associated with particularly high levels of genotoxic stress which need to be countered by powerful DNA repair and response mechanisms. These mechanisms maintain germination potential but also play a vital role in preservation of the genetic material transmitted between generations within the embryo genome. As such, DNA repair and response factors represent promising targets for the genetic improvement of crop germination performance in the field, in particular under stress conditions. Quantification of DNA damage levels or repair factors which are highly conserved across plant species, could also provide early and sensitive predictive markers for the evaluation of seed lot deterioration. Understanding genome maintenance mechanisms in seeds will be fundamental for the prediction and improvement of germination to help us meet major global challenges on the road ahead.

## Author Contributions

WW, CB, and CW conceived and wrote the review.

## Conflict of Interest Statement

The authors declare that the research was conducted in the absence of any commercial or financial relationships that could be construed as a potential conflict of interest.

## Abbreviations

AP: apurinic
ATM: ataxia telangiectasia mutated
ATR: ATM and RAD3-related
BER: base excision repair
DDR: DNA damage response
DSB: double-strand break
HR: homologous recombination
NHEJ: non-homologous end-joining
PARP: poly (ADP-ribose) polymerase
PCD: programmed cell death
QTL: quantitative trait locus
RAM: root apical meristem
ROS: reactive oxygen species
SMR: Siamese related
SSB: single-strand break
8-oxoG: 8-oxoguanine.
